# Orexin receptor antagonist-induced sleep does not impair the ability to wake in response to emotionally salient acoustic stimuli in dogs

**DOI:** 10.3389/fnbeh.2014.00182

**Published:** 2014-05-16

**Authors:** Pamela L. Tannenbaum, Joanne Stevens, Jacquelyn Binns, Alan T. Savitz, Susan L. Garson, Steven V. Fox, Paul Coleman, Scott D. Kuduk, Anthony L. Gotter, Michael Marino, Spencer J. Tye, Jason M. Uslaner, Christopher J. Winrow, John J. Renger

**Affiliations:** ^1^Department of In Vivo Pharmacology, Merck Research LaboratoriesWest Point, PA, USA; ^2^Department of Neuroscience, Merck Research LaboratoriesWest Point, PA, USA; ^3^Department of Medicinal Chemistry, Merck Research LaboratoriesWest Point, PA, USA

**Keywords:** orexin, DORA, hypocretin, sleep, arousal, auditory discrimination

## Abstract

The ability to awaken from sleep in response to important stimuli is a critical feature of normal sleep, as is maintaining sleep continuity in the presence of irrelevant background noise. Dual orexin receptor antagonists (DORAs) effectively promote sleep across species by targeting the evolutionarily conserved wake-promoting orexin signaling pathway. This study in dogs investigated whether DORA-induced sleep preserved the ability to awaken appropriately to salient acoustic stimuli but remain asleep when exposed to irrelevant stimuli. Sleep and wake in response to DORAs, vehicle, GABA-A receptor modulators (diazepam, eszopiclone and zolpidem) and antihistamine (diphenhydramine) administration were evaluated in telemetry-implanted adult dogs with continuous electrocorticogram, electromyogram (EMG), electrooculogram (EOG), and activity recordings. DORAs induced sleep, but GABA-A modulators and antihistamine induced paradoxical hyperarousal. Thus, salience gating studies were conducted during DORA-22 (0.3, 1, and 5 mg/kg; day and night) and vehicle nighttime sleep. The acoustic stimuli were either classically conditioned using food reward and positive attention (salient stimulus) or presented randomly (neutral stimulus). Once conditioned, the tones were presented at sleep times corresponding to maximal DORA-22 exposure. In response to the salient stimuli, dogs woke completely from vehicle and orexin-antagonized sleep across all sleep stages but rarely awoke to neutral stimuli. Notably, acute pharmacological antagonism of orexin receptors paired with emotionally salient anticipation produced wake, not cataplexy, in a species where genetic (chronic) loss of orexin receptor signaling leads to narcolepsy/cataplexy. DORA-induced sleep in the dog thereby retains the desired capacity to awaken to emotionally salient acoustic stimuli while preserving uninterrupted sleep in response to irrelevant stimuli.

## Introduction

Across the animal kingdom, the ability to awaken from sleep in response to salient signals (e.g., predator, fire alarm) and maintain sleep continuity in the presence of irrelevant background noise (e.g., environmental noise, traffic noise) is clearly important for both survival and healthy sleep. Auditory salience discrimination during normal sleep is the ability to discern between emotionally relevant versus irrelevant acoustic stimuli.

Classic studies in sleeping animals demonstrated that the threshold for awakening to emotionally relevant acoustic stimuli is much lower than the threshold for waking to an irrelevant auditory stimulus (Buendia et al., 1963; Dillon and Webb, 1965; Siegel and Langley, 1965; Van Twyver and Garrett, 1972; Langford et al., 1974; Halperin and Iorio, 1981; Coenen, 2010). Early laboratory studies involving the presentation of negatively conditioned acoustic stimuli, such as a tone associated with a shock, found animals more likely to awaken to these relevant conditioned versus neutral acoustic stimuli (Buendia et al., 1963; Van Twyver and Garrett, 1972; Halperin and Iorio, 1981). Such studies often invoked an underlying evolutionary drive to awaken in response to danger or predation signals with minimal disruption to sleep continuity in the presence of non-meaningful sounds, with the sleeping brain serving a sentinel function (see Snyder, 1966), as the ultimate benefit of such salience discrimination during sleep.

Human experimental paradigms have also demonstrated that sleeping people can exhibit an arousal response to salient acoustic stimuli over less salient stimuli, such as arousing to recordings of their own name over other names (Oswald et al., 1960; Langford et al., 1974; Coenen, 2010). Moreover, humans appear capable of differentiating acoustic stimuli at sub-arousal levels, as evidenced by oddball paradigm (Nordby et al., 1996), auditory evoked potential (Perrin et al., 1999), and neuroimaging (Portas et al., 2000) studies reporting that the sleeping brain can process acoustic stimuli as in wakefulness and distinguish emotionally relevant stimuli.

The threshold of arousal to acoustic signals in humans can be affected by central nervous system (CNS) depressants, such as benzodiazepines. In clinical studies, for example, flurazepam increased the arousal thresholds of subjects to both neutral (electronic tone) and emotionally salient (recording of subject’s name) acoustic stimuli (Mendelson et al., 1988). Similarly, triazolam impaired the ability of subjects to awaken to smoke-detector alarm signals (Johnson et al., 1987).

The orexin signaling pathway is conserved across mammalian species and plays an important role in regulating arousal and sleep (Sakurai et al., 1998; Gotter et al., 2012; Tsujino and Sakurai, 2013). Dual orexin receptor antagonists (DORAs), that block orexin receptors 1 and 2, have recently been developed and promote sleep by decreasing wake signaling and thereby enabling sleep (Brisbare-Roch et al., 2007; Winrow et al., 2011, 2012; Herring et al., 2012; Bettica et al., 2012b; Winrow and Renger, 2014). In contrast, the mechanism of action of the current standard of care for insomnia is mediated by positive allosteric modulators of inhibitory γ-aminobutyric acid (GABA)-A receptors (GABA-A receptor modulators). This mechanism involves sleep promotion through the suppression of CNS activity that can be associated with muscle relaxation, anxiolysis and amnesia due to relatively more widespread receptor expression (Nutt and Stahl, 2010; Tan et al., 2011). The question remains as to whether DORAs, which inhibit orexin-mediated arousal and wakefulness, promote sleep while maintaining the ability to salience gate relevant versus irrelevant acoustic stimuli by either waking or remaining asleep, respectively.

The present study used an auditory salience gating conditioning paradigm to investigate whether DORA-induced sleep preserved the ability of dog to wake in response to emotionally salient acoustic stimuli but remain asleep when exposed to irrelevant stimuli. Electronically generated acoustic stimuli were classically conditioned using either food reward and positive attention (salient) or nothing (neutral). Once conditioned, the acoustic stimuli alone were presented at times corresponding to maximal compound exposure or at the same time during natural (vehicle) sleep, and sleep/wake stages were assessed through continuous telemeterized electrocorticogram (ECoG), electromyogram (EMG), electrooculogram (EOG), and activity recordings.

Dogs were chosen to address salience gating of acoustic stimuli during DORA-induced sleep because their sleep and wake are more consolidated than preclinical rodent models, they do not exhibit sleep torpor, and they possess extremely sensitive auditory discrimination capacity. In addition, orexin signaling clearly plays a key role in the regulation of sleep in dogs as chronic loss of orexin signaling due to genetic mutations in orexin receptor 2 causes canine narcolepsy (Lin et al., 1999; Nishino, 2007), while temporary acute pharmacological blockade of orexin signaling by DORA administration can induce sleep in dogs without signs of narcolepsy/cataplexy (Brisbare-Roch et al., 2007; Winrow et al., 2011, 2012).

Although DORAs produce a transient and reversible pharmacological blockade of orexin receptors, not a chronic loss of orexin signaling across the lifespan as in dog narcolepsy, we also assessed cataplexy with high-dose DORA administration in the standard canine Food Elicited Cataplexy Test (FECT; see Babcock et al., 1976). A preliminary FECT study was necessary to confirm that classical conditioning of an acoustic stimulus with a salient food reward would not potentially confound a study of awakening from sleep, as strong emotional salience as seen with food anticipation is a primary trigger of cataplexy in orexin-deficient narcoleptic dogs (Babcock et al., 1976; Kushida et al., 1985; Nishino et al., 1997).

Finally, since the veterinary literature qualitatively cites case studies of accidental GABA-A receptor modulator and antihistamine ingestion causing paradoxical hyperarousal in healthy dogs (Barnett et al., 1984; Bertini et al., 1995; Richardson et al., 2002; Wismer, 2002; Herron et al., 2008; Lancaster et al., 2011), we quantitatively pre-tested several representative drugs via polysomnography (PSG) to determine whether we could evaluate different pharmacological hypnotic mechanisms in the salience gating arousal paradigm.

In response to the salient conditioned acoustic stimuli, dogs in the present study awoke completely from vehicle and orexin-antagonized sleep across all sleep stages but rarely awoke to the neutral stimuli.

## Materials and methods

### Animal subjects

Adult male and female Beagle dogs (2–14 years old, 8–17 kg) were singly housed for the study duration under standard indoor laboratory conditions of controlled temperature, humidity, and lighting (12-h light:12-h dark), with a single mid-day feed. Water was provided *ad libitum*. All studies were conducted in accordance with the Merck Institutional Animal Care and Use Committee and the National Research Council’s Guide for the Care and Use of Laboratory Animals.

### Drugs and pharmacokinetics

DORA-22 and DORA-12 were synthesized at Merck as previously described (Cox et al., 2010; Coleman et al., 2012; Winrow et al., 2012). Representative GABA-A receptor modulators and a histamine inverse agonist, which tend to promote sleep in most species but have paradoxical hyperarousal effects in dogs according to qualitative reports in the canine veterinary literature (Barnett et al., 1984; Bertini et al., 1995; Richardson et al., 2002; Wismer, 2002; Herron et al., 2008; Lancaster et al., 2011; Giorgi et al., 2012), were also evaluated in the sleep studies as potential control sleep-inducing substances for the salience arousal studies. Diazepam was purchased from Sigma-Aldrich, eszopiclone from Sunovion Pharmaceuticals, zolpidem from Teva Pharmaceuticals, and diphenhydramine from Henry Schein. DORA-22 (0.3, 1, 5 mg/kg), DORA-12 (1 mg/kg), and eszopiclone (0.5, 5, 12 mg/kg) were administered orally by gavage in 20% vitamin E tocopherol polyethylene glycol succinate (TPGS); diazepam (1 mg/kg) and zolpidem (1 mg/kg) were administered orally by gavage in 0.5% carboxymethylcellulose; and diphenhydramine (4 mg/kg) was administered in saline (intramuscularly).

Pharmacokinetic assessments were performed to determine plasma levels of the DORAs in study dogs approximately 1 week after studies were completed to avoid confounding EEG and behavioral assessments; DORAs were orally dosed in fasted dogs as detailed above. Blood was collected from the femoral artery in the presence of EDTA at 0.25, 0.5, 1, 2, 4, 6, and 24 h post-dose, and the resulting plasma was stored frozen at −20°C. Plasma concentrations were determined by protein precipitation followed by liquid chromatography–tandem mass spectrometry in the positive ion mode using atmospheric pressure chemical ionization. The lower limit of quantitation for both compounds was 0.002 uM.

### Dog PSG and sleep staging

Dogs were implanted with subcutaneous telemetric devices (D70-EEE; Data Sciences International) to record simultaneously ECoG, EMG, EOG and locomotor activity using the surgical procedure described previously (Winrow et al., 2011, 2012). All implant surgeries were completed at least 6 months prior to study initiation, with a minimum of 2 weeks after the most recent subcutaneous battery-replacement procedure.

Sleep stages were evaluated using both continuous hand-scoring (2–4 h surrounding dosing) and a customized version of the sleep algorithm Somnologica (Embla Systems) based on a combination of ECoG, EMG, and EOG activity and movement within the field of the radiofrequency receiver, as described previously (Winrow et al., 2011, 2012). Briefly, ECoG/EMG/EOG/locomotion data were used to characterize four sleep-wake stages in the dogs: active wake, slow wave I sleep (lighter non-rapid eye movement [REM] sleep), delta II sleep (deep non-REM sleep), and REM sleep. Sleep architecture data were evaluated in 30-s epochs and then averaged into 30-min bins across 24 h (sleep studies only).

### Sleep-wake study design

All sleep-wake studies used a within-subject cross-over design (vehicle × drug) with telemetric PSG recordings 24 h/day. In DORA sleep studies (*n* = 6 male dogs/study; 5 days vehicle × 5 days drug), dogs were dosed at Zeitgeber time (ZT) 03:00 (daytime studies) to represent the active period and at 1 h prior to lights out (ZT 11:00 for nighttime studies, DORA-22 only) to align peak efficacy during lights-out without waking the dogs for dosing. In the GABA modulator/antihistamine sleep studies (*n* = 6–7, 1–3 days vehicle × 1–3 days drug), dogs were dosed at ZT 03:00.

### Food Elicited Cataplexy Tests (FECT)

Daytime FECTs (*n* = 11; 6 males, 5 females) (Babcock et al., 1976; Kushida et al., 1985; Nishino et al., 1997) with simultaneous video and ECoG/EMG/EOG recordings were conducted with vehicle (*n* = 2 tests/dog) and DORA-12 (*n* = 2 tests/dog). DORA-12 has been previously shown to promote sleep, and the dose selected robustly decreases wakefulness in dogs (>100-fold minimum efficacious sleep dose; see below). FECTs were conducted approximately 45–60 min post-dose in an empty room to which animals were habituated prior to testing, as described in the FECT literature (Babcock et al., 1976; Kushida et al., 1985; Nishino et al., 1997). Briefly, each dog was singly released into the test room and remotely monitored while consuming 10 approximately 1 inch^2^ balls of wet dog food (Pedigree® chunk beef), which were evenly spaced one foot apart down the middle of a clean disposable absorbent pad placed on the floor. Video and ECoG/EOG/EMG recordings were evaluated for any signs of cataplexy (e.g., collapse, EMG attenuation, sudden-onset REM sleep; see Kushida et al., 1985) and the time to consume all 10 balls of food.

### Salience arousal studies

Dogs (*n* = 6–8; 3–4 males, 3–4 females) were housed in two separate rooms of 3–4 dogs each, equipped with a central auditory speaker and video camera. Two novel, electronically generated (Spike2 v5.2.1, Cambridge Electronic Design) distinct acoustic stimuli of 700 Hz and 1000 Hz (50 dB, 3-s duration) were selected based on the criteria that the tones’ decibel levels varied no more than 3 dB between the interiors of each individual dog run. The ambient noise level in the dog room was approximately 50 dB at all times, without barking. The tones representing the neutral stimulus (neutral) and salient positively reinforced, classically conditioned stimulus (salient CS) were counterbalanced across the rooms (e.g., Room 1 dogs: neutral = 700 Hz, salient = 1000 Hz; Room 2 dogs: neutral = 1000 Hz, salient = 700 Hz).

**Figure 1:**
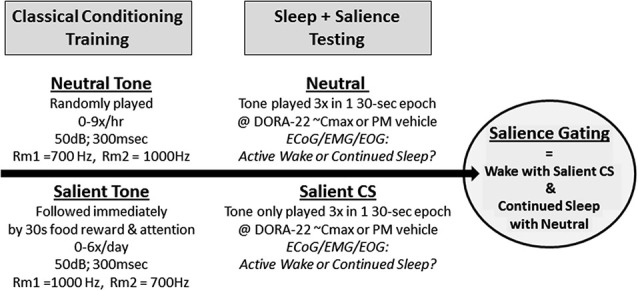
**Salience-gated arousability paradigm in dogs**. Dogs (*n* = 8) were classically conditioned to anticipate nothing (Neutral) or food reward with positive attention (Salient) following a 300 ms acoustical stimulus. During salience testing, only the Neutral or Salient Conditioned Stimulus (CS) tone were presented to sleeping dogs (2 tests/dog/condition); no room entry or reward was given. Telemeterized polysomnography (PSG) was used to quantify Active Wake or continued sleep in response to the differently salienced stimuli.

Conditioning to the neutral stimuli occurred in the 6 weeks prior to and for the duration of the arousal studies. During this time, the neutral stimulus was played in the dog rooms at randomly generated times in clusters of 3 tones/30 s (parallels 30 s epoch for sleep stage scoring), with an average of 3 clusters (9 tones) per hour, 0–15 h/day (maximum of 3 h/day during lights out). Dogs were monitored by video (i.e., no behavioral reaction) and by PSG (i.e., sleep fragmentation parameters demonstrating equivalence of the neutral tone with the absence of an acoustic stimulus; data not shown) prior to positive conditioning of the salient tone and during the study to confirm that the neutral stimulus was indeed neutral. See Figure 1. For 3 weeks prior to arousal study initiation, the salient acoustic stimulus was immediately reinforced with bites of wet food (Pedigree® chunk beef) and positive attention, see Figure 1. Food and attention were delivered by assistants who entered the room immediately upon salient tone delivery; assistants wore visually distinct lab coats and had never been involved in the dogs’ dosing, blood sampling, surgery, or cage cleaning. The salient tone was played randomly in clusters of 1–3 tones/3 min, each followed by 30 s of reinforcement, 0–2 times/day (daytime and lights out). Approximately 1 h prior to a salient tone reinforcement session (0–2 times/week), the dogs were dosed with saline to ensure that dosing did not inadvertently become a contextual cue during the actual study. Positive reinforcement of the salient stimulus continued 1–3 times/week for the study duration. Prior to and during the arousal study, dogs were monitored by video to confirm that the salient CS was indeed salient (e.g., the tone elicited immediate visual orientation to the human entry door, barking, and jumping).

During the arousal study trials, only the neutral and salient acoustic tones were played; no reinforcement followed the salient stimulus (Figure 1). Continuous video and ECoG/EMG/EOG recordings occurred before, during, and after stimulus presentation. Animals were dosed with either vehicle (night testing only) or DORA-22 (0.3, 1, 5 mg/kg; doses were randomized) approximately 30–60 min prior to a stimulus presentation, with 0–1 test per day. Dogs received both one neutral 3-tone/30-s cluster and one CS 3-tone/30-s cluster during each day’s test (counterbalanced order, separated by 15–30 min). Tone presentation was simultaneously digitally marked on the PSG recording trace using DSI software. Each dog was ultimately tested twice per DORA-22 dose: once with the neutral stimulus first, and once with the CS stimulus first. Dogs received four counterbalanced vehicle-only night control tests. Vehicle treatment was not assessed in the daytime studies because the dogs were not typically asleep.

Data from individual tests were excluded if the dog’s PSG recordings did not confirm sleep during the 2 min immediately preceding the stimulus tone. Arousal was defined as at least a single 30-s epoch of PSG-defined “Active Wake” beginning with the epoch containing the three stimulus tones.

### Data analysis

Overall, the cross-over design studies utilized either a repeated measures analysis of variance or paired two-tailed *t*-tests. All data are reported as the mean per animal (across days or tests) and the variability (standard error of the mean).

An independent mixed-model analysis was applied for the multi-day 24-h sleep architecture results for each 30-min time bin versus vehicle averaged per animal across days, as described previously (Winrow et al., 2011). In the PSG comparison studies, the mean cumulative time in active wake for each dog during the first 2 h after compound dosing was expressed as the percentage change from vehicle administration. A two-tailed population *t*-test was used to assess the change in wake for each compound compared with 0 (no change in wake from vehicle).

## Results

### DORA pharmacokinetics

DORA-12, used to maximize exposure for the FECT, at 1 mg/kg produced peak plasma concentration levels (C_max_) of 1.63 μM ± 1.3 with a 24-h drug exposure value (area under the curve; AUC) of 9.52 μMh. DORA-12 plasma exposure peaked (T_max_) at 0.5 h ± 0.0. During the FECT studies, the plasma levels at 1–2 h post-dose were approximately 1.5 μM, which is >100-fold higher than minimum efficacy exposure levels tested for this compound to induce sleep in adult dogs (an approximately 30% decrease in wake was observed with C_max_ = 0.014 μM ± 0.008 and AUC = 0.07 μMh).

DORA-22, used in the arousal studies, exhibited dose-responsive plasma pharmacokinetic levels across 0.3 mg/kg (C_max_ = 0.26 μM ± 0.07, AUC = 0.63 μMh ± 0.1), 1 mg/kg (C_max_ = 0.91 μM ± 0.08, AUC = 3.87 μMh ± 0.2), and 5 mg/kg (C_max_ = 3.02 μM ± 0.5, AUC = 12.65 μMh ± 2.5), a 20-fold range of exposure. T_max_ values were relatively constant across doses: 0.7 h, 0.7 h, and 0.5 h, respectively, which approximately corresponded to the time of acoustic stimulus presentation in the salience arousal studies.

### Sleep-wake

**Figure 2:**
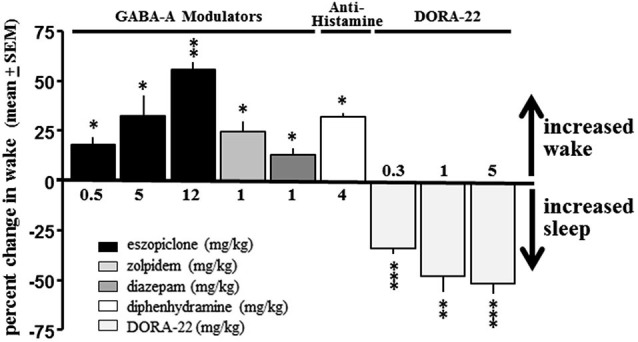
**DORA-22 promotes sleep in beagle dogs**. GABA-A receptor modulators (eszopiclone and zolpidem) and antihistamine (diphenhydramine) produce paradoxical hyperarousal but DORA-22 induces sleep in dogs. Only DORA-22 was thereby assessed for salience-gated arousability and compared to unmedicated healthy sleep in dogs. Data expressed as mean percentage change ± SEM from vehicle during the first 2 h post-dose during the daytime active period. * *p* < 0.05; ** *p* < 0.01; *** *p* < 0.001.

No GABA-A receptor modulator tested produced a sleep effect in dogs when administered during the day; diazepam, eszopiclone and zolpidem all had significant paradoxical hyperarousing effects, as did the antihistamine diphenhydramine (Figure 2). These results are consistent with *ad hoc* reports in the canine veterinary literature (Barnett et al., 1984; Bertini et al., 1995; Richardson et al., 2002; Wismer, 2002; Herron et al., 2008; Lancaster et al., 2011; Giorgi et al., 2012). However, daytime administration of DORA-22 significantly decreased active wake (Figure 2). Vehicle-treated nighttime sleep in dogs, therefore, was selected as the sole sleep comparator for DORA-22 sleep in the salience arousal paradigm as the other potential hypnotics did not produce sleep in the dog.

**Figure 3:**
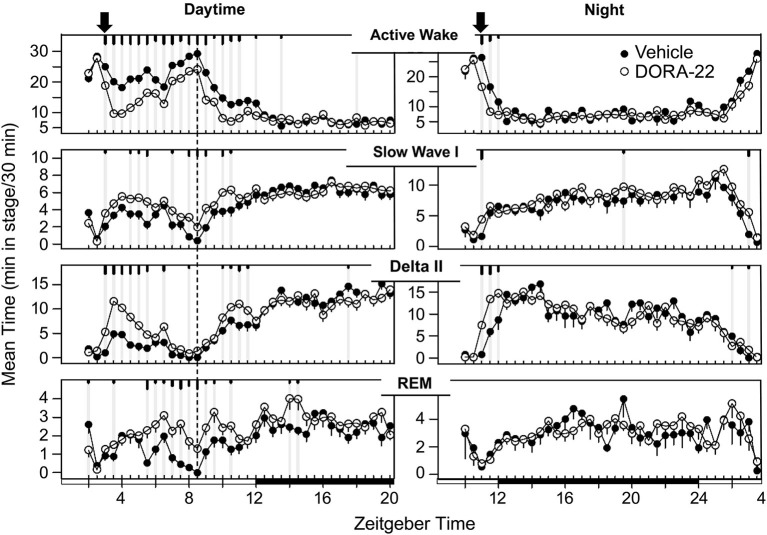
**Dog sleep architecture with DORA-22 dosed during daytime (left) or prior to night lights-out (right)**. DORA-22 (open circles; 5 mg/kg) and vehicle (20% Vitamin E TPGS, closed circles) were administered to the same dogs (*n* = 6) in a balanced cross-over design. Data are presented as mean per dog over 5 days of consecutive treatment per 30-min epoch ± SEM. Arrows, dosing; dashed line, feeding (daytime); gray vertical lines and tic marks (short, medium, long: *p* < 0.05; *p* < 0.01; *p* < 0.001, respectively).

The sleep architecture underlying the decrease in active wake with administration of the DORA-22 5 mg/kg (*n* = 6) dose used in the daytime and nighttime sleep trials is presented in Figure 3. During daytime administration, DORA-22 significantly decreased active wake, and significantly increased slow wave sleep, delta sleep, and REM, all relative to the vehicle control, to approximately nighttime levels. DORA-22 administered 1 h prior to lights off (night period) decreased active wake and increased sleep at the time of dosing; after lights off, DORA-22 sleep paralleled vehicle nighttime sleep architecture.

### Food Elicited Cataplexy Test

**Figure 4:**
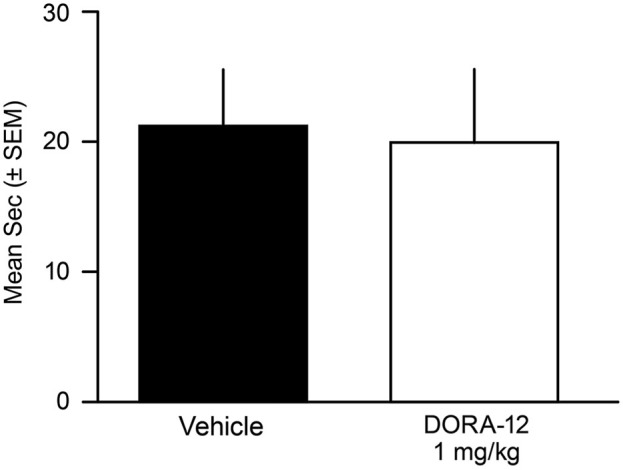
**Time to complete Food Elicited Cataplexy Test (FECT)** **was not affected by high-dose DORA-12.** Dogs showed no physical signs of cataplexy (see Babcock et al., 1976) and there was no difference in the mean duration (seconds/dog/2 tests ± SEM) to complete FECT on vehicle (20% Vitamin E TPGS; black) compared to the same dogs (*n* = 11) with high-dose DORA-12 (1 mg/kg, 100x minimum efficacious sleep dose; white) tested at peak exposure.

No male or female dog showed any behavioral or EMG-characteristic signs of cataplexy during the FECT study; the dogs were continuously in active wake (by video and PSG assessment) through the completion of all food consumption. Overall, the duration to complete the FECT did not differ between the vehicle and drug tests for all dogs (*t*_(10)_= 0.41, *p* = 0.69) (Figure 4). The percentage change per dog between time to complete the FECT with vehicle and DORA-22 did not differ from zero (*t*_(10)_ = 0.20, *p* = 0.86).

### Salience arousal studies

**Figure 5:**
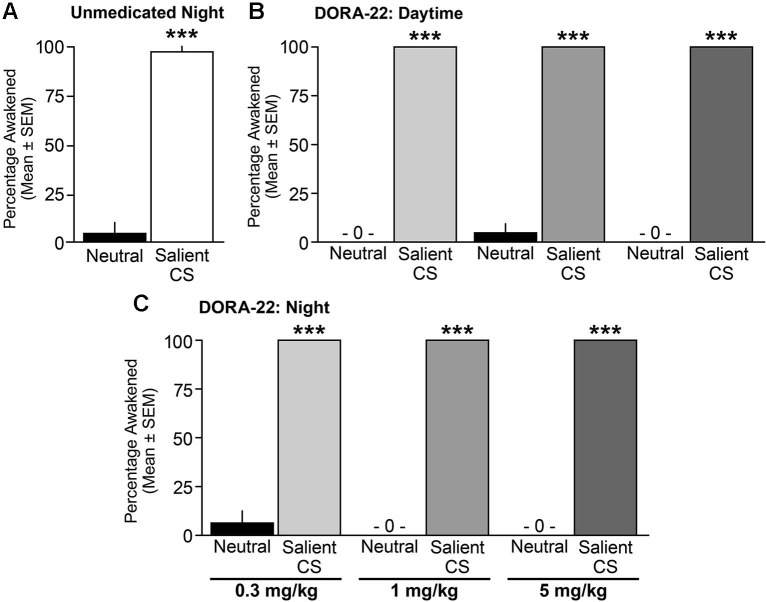
**Dogs awoke to the conditioned salient stimulus and slept through the neutral stimulus**. Percentage of dogs (*n* = 6–8) aroused into Active Wake during **(A)** unmedicated (20% Vitamin E TPGS vehicle) night sleep, **(B)** DORA-22-induced daytime sleep, and **(C)** DORA-22-induced night sleep with the neutral acoustical stimulus and salient conditioned stimulus (CS). Mean percentage per dog across 2 tests/condition ± SEM. ***p < 0.0001.

When tested during the nighttime (lights-out) period, vehicle-treated male and female dogs awoke from sleep significantly more often to the salient CS tone than to the neutral tone (*t*_(16)_ = 8.85, *p* < 0.0001; Figure 5A). With regard to DORA-22-induced daytime sleep, dogs woke significantly more to the salient CS tone than to the neutral tone for all DORA-22 doses tested (*F*_(1,14)_ = 2209, *p* < 0.0001; each dose neutral-salient *post-hoc* comparison *p* < 0.0001; Figure 5B). In the night sleep trials, dogs again woke significantly more from DORA-22-induced nighttime sleep in response to the salient CS tone than to the neutral tone for all doses tested (*F*_(1,14)_ = 2209, *p* < 0.0001; each dose neutral-salient *post-hoc* comparison *p* < 0.0001; Figure 5C). Although each test was comprised of a cluster of 3 tones within a 30-s epoch, all arousals occurred in response to the first tone. There was no overall dose effect or interaction between dose and acoustic stimulus.

Across all studies, sleep stage did not predict whether a dog responded or did not respond to an acoustic stimulus. In response to the salient CS tone, dogs awoke from all sleep stages (Table 1). Dogs remained asleep when the neutral tone was presented at all sleep stages, although there was a modest trend in the sleep architecture for the animals to shift from a deeper sleep stage to a lighter sleep stage (Table 1). The single occurrence of continued sleep in response to the salient CS tone (vehicle, night test) was during slow wave I sleep. Three cases of arousal to the neutral stimulus (one each with vehicle and DORA 0.3 mg/kg, night test, and one with DORA 1 mg/kg, daytime test) occurred during delta II or REM sleep.

**Table 1:** **Arousal or continued sleep in response to acoustic stimuli by sleep architecture stage**.

	**Slow wave I**	**Delta II**	**REM**
**Salient conditioned stimulus – awoke†**	31.7%	58.5%	9.8%
**Immediately before neutral**	43.5%	46.5%	10.0%
**Neutral stimulus – continued sleep‡**	52.5%	40.0%	7.5%

The distribution is presented as the percentage of all tests per condition and result.

†One vehicle-treated dog did not wake with the salient conditioned stimulus (slow wave I sleep);

‡one vehicle- and two DORA-treated dogs were aroused with the neutral tone from delta II (*n* = 2) and REM sleep (*n* = 1).

**Figure 6:**
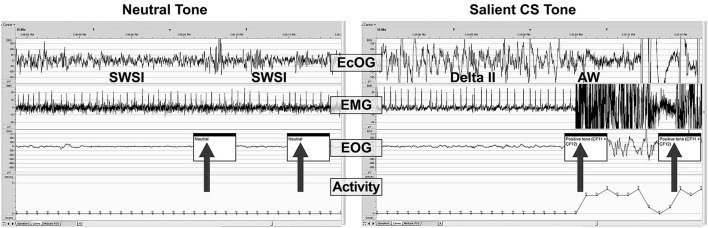
**A sample PSG trace during DORA-22 presentation of a neutral (left) and salient conditioned (right) stimulus**. Thirty-second sample of PSG (ECoG, EMG, EOG, and locomotor activity) traces for a dog (subject 10-0451) approximately 45 min after receiving DORA-22 1 mg/kg oral daytime dosing in response to neutral (left) and salient conditioned (CS, right) acoustic stimuli presentation (arrows). Note that the dog remains asleep during the neutral tone (only ECoG perturbation), but immediately awakens into Active Wake with the salient CS tone. The first 2 tones of a 3-tone/30-s epoch are shown. AW, active wake; ECoG, electrocorticogram; EMG, electromyogram; EOG, electrooculogram; SWSI, slow wave I sleep; delta II, delta II sleep.

Figure 6 shows examples of PSG traces from a single dog treated with DORA-22 1 mg/kg during the day, and exposed to neutral and CS acoustic stimuli. In both examples, the animal is asleep prior to the first tone presentation. With the neutral tone presentation, the dog remains in slow wave I sleep. Note that a brief perturbation may be seen in the ECoG trace at the time of the first neutral tone, yet the animal remains asleep. In contrast, with the presentation of the first salient CS tone, the dog immediately transitions from delta II sleep to active wake, as reflected in the ECoG, EMG, EOG, and locomotor traces.

## Discussion

The present study used an auditory salience gating conditioning paradigm in dogs to investigate whether DORA-induced sleep preserved natural sleep’s ability to awaken appropriately to emotionally salient acoustic stimuli but remain asleep when exposed to irrelevant stimuli. The PSG data confirmed dose-responsive sleep-promoting effects of orexin antagonism in dogs. When administered during the daytime or just prior to nighttime lights off, DORA treatment did not impair the ability to respond selectively to emotionally salient acoustic stimuli: at all doses and times, dogs aroused to emotionally salient acoustic stimuli but slept through neutral acoustic stimuli. The dogs’ salience gating ability during DORA-induced sleep was identical to salience gating during natural (vehicle) nighttime sleep.

Dogs exhibit a conserved, translatable, and reliable orexin antagonist-associated sleep response (Brisbare-Roch et al., 2007; Winrow et al., 2011, 2012) not observed with GABA-A modulator hypnotics or an antihistamine known to have sedating properties in humans. The present study quantitatively confirmed dog paradoxical hyperarousal reported with accidental ingestion of these agents (Barnett et al., 1984; Bertini et al., 1995; Richardson et al., 2002; Wismer, 2002; Herron et al., 2008; Lancaster et al., 2011; Giorgi et al., 2012). In contrast, daytime and before nighttime DORA administration significantly and dose-responsively decreased the amount of time spent in active wake and increased slow wave, delta and REM sleep to parallel normal nighttime sleep architecture distribution and percentages, consistent with the effects of structurally diverse DORAs reported by a number of groups in dogs and other sleep models and in humans (Brisbare-Roch et al., 2007; Winrow et al., 2011, 2012; Bettica et al., 2012a, b; Fox et al., 2013). DORA administration may thus represent the only non-anesthesia pharmacological method currently known to reliably induce sleep in dogs. However, due to the lack of somnolence-inducing effects of GABA-A receptor modulators or antihistamine in dogs, this model was unable to compare directly salience gating during sleep induced by these classes of drugs with natural and DORA-induced sleep. The mechanism underlying these paradoxical effects in canines are currently unknown, but may involve dog-dependent differences in the balance of GABA-A and orexin-mediated signaling in promoting sleep and arousal, respectively (Saper et al., 2005). Additional studies in other mammalian sleep models and humans may allow for such comparisons of the differences in salience gating between DORA and GABA-A modulators, as well as potentially elucidate the neural circuits underlying differences among species.

DORA administration was negative in FECT, and the time required to complete the FECT for vehicle- and DORA-treated dogs in this study was consistent with that reported for normal dogs (within 45 s; Babcock et al., 1976). The lack of effect of DORA administration in the FECT suggests that acutely antagonizing an intact orexin system in normal dogs differs from the pathological chronic condition of genetic orexin depletion in narcoleptic dogs. These data are consistent with previous studies demonstrating that orexin antagonism does not recapitulate the phenotypes of genetic orexin deficiency (Chemelli et al., 1999; Lin et al., 1999; Brisbare-Roch et al., 2007; Winrow et al., 2011, 2012; Mang et al., 2012). Perhaps even more strikingly along this acute versus chronic orexin-depletion dichotomy, the type of strong emotionally salient stimuli—food—that typically induces sleep-like cataplexy in orexin-deficient narcoleptic dogs during the FECT is the same CS association that caused DORA-treated dogs to awaken from sleep in the present study.

It has been established that the emotional significance of a stimulus is associated with the ability to induce an arousal response from natural sleep, as demonstrated by early sleep experiments in rodents (Van Twyver and Garrett, 1972), cats (Buendia et al., 1963; Siegel and Langley, 1965), and humans (Oswald et al., 1960; Langford et al., 1974). Consistent with the historical literature, the DORA- and vehicle-treated dogs in this study awoke completely in response to the emotionally salient conditioned tone presentation but were not aroused by similar tones played randomly without conditioning. These results clearly indicate that neither discrimination between neutral and salient stimuli nor the arousal in response to salient stimuli is dependent upon orexin, but likely a downstream mechanism. More work will need to be done to investigate the mechanism of acute arousal, but potential explanations beyond the scope of the current work include the disinhibition of GABA tone, downstream pathways affecting histamine release or other, yet to be defined pathways affecting CNS arousal.

Some studies in other sleep models have reported differences in arousability from different sleep stages, and while REM sleep was originally hypothesized to be associated with increased vigilance, this has not been confirmed experimentally (Siegel and Langley, 1965; Snyder, 1966). In the current dog studies, the PSG analyses indicated that vehicle- and DORA-treated dogs were similarly able to awaken from all sleep stages in response to the conditioned acoustic stimulus, without a predictive effect of sleep stage on arousability. It remains possible, however, that a larger sample size may reveal salience arousability differences among sleep stages.

In working with dogs, a specific decision was made in the present study to use a positively conditioned salient stimulus instead of a negatively CS (e.g., shock), as has often been used in the salience arousal literature. The orexin peptides, particularly Orexin-A, are known to be associated with increased feeding behaviors in animals, whereas orexin antagonism is reported to decrease eating (Sakurai et al., 1998; Haynes et al., 2000; Rodgers et al., 2000; Tsujino and Sakurai, 2013). Accordingly, we risked having the DORA effects blunt the salience of a food-associated CS. Although we cannot directly address any modulating impact of DORAs on the salience of the food-conditioned stimulus, at the very least the CS was sufficiently salient to distinguish it from the neutral cue. Video recordings during the arousal testing subjectively confirmed animal excitation upon presentation of the salient stimulus during both the vehicle and DORA trials. Meanwhile, the predominant use of negatively conditioned stimuli in studies of salience gated arousal in natural sleep in the historical animal literature (Buendia et al., 1963; Siegel and Langley, 1965; Van Twyver and Garrett, 1972) gives confidence that negative stimuli may also be associated with differential increases in arousal during DORA-induced somnolence.

Further investigations of salience discrimination during DORA-induced sleep in additional models and humans are warranted, as this new class of insomnia agent may have the potential to allow patients to sleep undisturbed by benign stimuli (e.g., traffic noise, partner snoring) but not miss responding to important signals (e.g., smoke detector, intruder).

## Conclusion

As with natural sleep, dogs administered DORAs retained the ability to distinguish between irrelevant auditory sounds and important cues and responded by remaining asleep or waking up from all sleep stages, respectively. The results from this study further reinforce the notion that acute DORA treatment differs from the endogenous developmental low orexin levels in narcoleptic dogs in that DORA treatment resulted in normal sleep architecture, lack of signal in the FECT, and response to an emotionally salient stimulus with immediate awakening instead of cataplexy. It is perhaps fitting, therefore, that while the role of the orexin peptide was originally elucidated in dogs, DORA-induced sleep may be the only reliable method aside from anesthesia to potentially come full circle and pharmacologically induce sleep in dogs. Finally, maintaining salience-specific arousal is yet another way DORAs have the potential to differentiate from existing insomnia agents.

## Conflict of interest statement

All authors are employees of Merck and Co., Inc.
